# A Multi-task Learning Model for Daily Activity Forecast in Smart Home

**DOI:** 10.3390/s20071933

**Published:** 2020-03-30

**Authors:** Hong Yang, Shanshan Gong, Yaqing Liu, Zhengkui Lin, Yi Qu

**Affiliations:** 1 School of Information Science & Technology, Dalian Maritime University, Dalian 116026, China; yanghong@dlmu.edu.cn (H.Y.); gongshanshan66@dlmu.edu.cn (S.G.); zhengkuilin@dlmu.edu.cn (Z.L.); quyi@dlmu.edu.cn (Y.Q.); 2 Artificial Intelligence Key Laboratory of Sichuan Province, Sichuan University of Science and Engineering, Zigong 643000, China

**Keywords:** multi-task learning, deep learning, daily activity forecast, smart home

## Abstract

Daily activity forecasts play an important role in the daily lives of residents in smart homes. Category forecasts and occurrence time forecasts of daily activity are two key tasks. Category forecasts of daily activity are correlated with occurrence time forecasts, however, existing research has only focused on one of the two tasks. Moreover, the performance of daily activity forecasts is low when the two tasks are performed in series. In this paper, a forecast model based on multi-task learning is proposed to forecast category and occurrence time of daily activity mutually and iteratively. Firstly, raw sensor events are pre-processed to form a feature space of daily activity. Secondly, a parallel multi-task learning model which combines a convolutional neural network (CNN) with bidirectional long short-term memory (Bi-LSTM) units are developed as the forecast model. Finally, five distinct datasets are used to evaluate the proposed model. The experimental results show that compared with the state-of-the-art single-task learning models, this model improves accuracy by at least 2.22%, and the metrics of NMAE, NRMSE and R^2^ are improved by at least 1.542%, 7.79% and 1.69%, respectively.

## 1. Introduction

One of goals of smart home development is to provide residents a comfortable and safe living space [1,2]. Smart homes are expected to be able to prompt or warn residents about their health condition [3,4,5,6] by recognizing and forecasting upcoming daily activity [7,8,9,10]. As far as daily activity forecast is concerned, category forecasting and occurrence time forecasting of daily activities are two key tasks. Category forecasts are devoted to forecasting which daily activity is about to occur. Occurrence time forecasts are devoted to forecasting when a given daily activity occurs. 

So far, category forecasts and occurrence time forecasts of daily activity have been explored separately rather than as a whole [11,12,13,14]. Daily activity forecasts are usually separated into several independent sub-tasks, and then the results of sub-tasks are combined. However, these serial and separate approaches perform not well enough [15]. 

To improve the performance of daily activity forecasting, this paper proposes a forecast model based on multi-task learning. The proposed model assumes that the category forecasts and occurrence time forecasts of daily activities are related to each other. Combining the two forecast tasks into a network model can not only ensure their co-training, but also promote the generalization and performance of the model by weighing the training information in the two related tasks. 

The key contributions of this paper are:

(1) A daily activity forecast model based on multi-task learning is proposed. The proposed model decomposes the features of recent sensor events, and then constructs the forecast model from these generated features using multi-task learning technology.
(2) The proposed model is evaluated in detail on five distinct datasets.

This paper is organized as follows: Section 2 reviews related work. Section 3 introduces the problem formulation. Section 4 describes the datasets used. Section 5 describes the forecast model of multi-task learning in hybrid networks. Section 6 provides regression and classification tasks metrics. Section 7 discusses different task loss weights and sliding window sizes, further validates the proposed approach and analyzes the results. Finally, Section 8 concludes this paper with a brief summary of our findings.

## 2. Related Work

For category forecasting of daily activity, Gopalratnam et al. proposed a probabilistic method based on an improved Markov model [16] without considering the uncertainty of daily activities. Alam et al. employed the SPEED model to forecast daily activity categories via analyzing the sequences of daily activities that were occurring [17]. This method was further refined by an All Discoverable Episodes (SPADE) model [12]. Channe et al. used an Apriori model to mine frequent control sequences in sensor data [18]. Similarly, due to the chaos of the control sequence in different time periods, the performance of the prediction results was poor. Neural networks were also used in sequence prediction research and achieved improved performance. Sungjoon et al. used a hybrid network framework to predict various daily activities [19]. A recursive neural network (RNN) [20,21,22,23,24] and LSTM network [13] were used in daily activity forecasting in an in-depth study. Although these neural networks improved the forecast performance to some extent, they only trained the category forecast model of daily activities and ignored the time information of the daily activities themselves. 

For occurrence time forecast of daily activity, popular models include the autoregressive moving average (ARMA) and autoregressive integrated moving average (ARIMA) [25]. Scellato et al. forecasted the timing and duration of daily activities by analyzing the average of data from previous similar sequences [26]. Rule-based models were employed for occurrence time forecasts, but these could not account for more complex daily activities [2,27]. A non-linear autoregressive network (NARX) was used to predict the start and end time of sensor activation, but it was not effective in the relevant prediction of daily activities [28]. Mahmud et al. forecasted the next daily activity occurrence time based on the Poisson process [29]. Similarly, Minor et al. independently trained a predictive regression model for occurrence time of specified daily activities based on additional feature sets [14,30]. Due to their accessibility limitations, it was not always feasible to add additional feature sets to the model. 

For daily activity forecasting, Nazerfard et al. used Bayes networks to forecast daily activity. Nazerfard et al. constructed a normal mixture model based on an expectation maximization (EM) algorithm to obtain the occurrence time range. Since the time forecast relied heavily on the activity label predicted in the previous step, error propagation easily occured [15]. The combination of LSTM and k-means was used to solve the prediction problem of the next sensor event, but they were essentially independent models for sensor and trigger time forecast [31]. To our best knowledge, all of prior forecast strategies dealt with a certain forecast task independently without the parallel training of the two tasks. Thus, the correlation information of the original related tasks was missing. 

Multi-task learning has replaced previously conventional independent learning with multiple related tasks. The aim was to improve the model generalization ability [32,33,34,35]. Neural network-based multi-task learning has been applied in many fields [36,37,38,39]. Long et al. added matrix priors into the full connection layer to learn the relationships between tasks. Due to the need for a predefined shared structure, there was an error bias for the new tasks [40]. Cross-stitch networks solvd problems without universality for multi-task network structures, but many parameters in the model were redundant [41]. Reference [42] was similar to [41] in essence, but the algorithm was relatively simple. Li et al. utilized a 3D CNN combined with multi-task learning to extract spatiotemporal features. Attention-based LSTM was then used for feature embedding, but the outliers were not handled effectively, which could affect the model performance [43]. According to the needs of each task, CNNs stochastic filter groups grouped the convolution kernel of each convolution layer [44]. There are some other networks such as branched multi-task networks [45], sluice networks [46] and learning sparse sharing [47] to address multiple task sharing issues, but it was difficult to train them due to the high complexity of the model. There are also low supervision [48] and self-supervised learning [49] which are used to do part-of-speech tagging or other issues in the NLP field. In the image application field, Yang et al. extended the model parameter division to obtain the correlation coefficient between shared parameters and tasks [50]. Reference [51] described a soft attention mask which learned jointly with features in the shared network to maximize the generalization of shared features in multiple tasks.

The proposed approach falls into the field of daily activities forecasting in smart homes. To our best knowledge, the state of the art has focused on either forecasting daily activities or forecasting the time when a given daily activity will occur. For the approach presented in this paper, multi-task learning is firstly employed to forecast daily activity. Compared with the state of the art, the proposed approach performs these two tasks as a whole. Based on the nature of multi-tasks, this paper presents a multi-task learning approach for daily activities forecast. The proposed approach features that each task forecast result learns mutually and iteratively in order to improve the forecast performance of each task.

## 3. Problem Formulation

**Figure 1:**
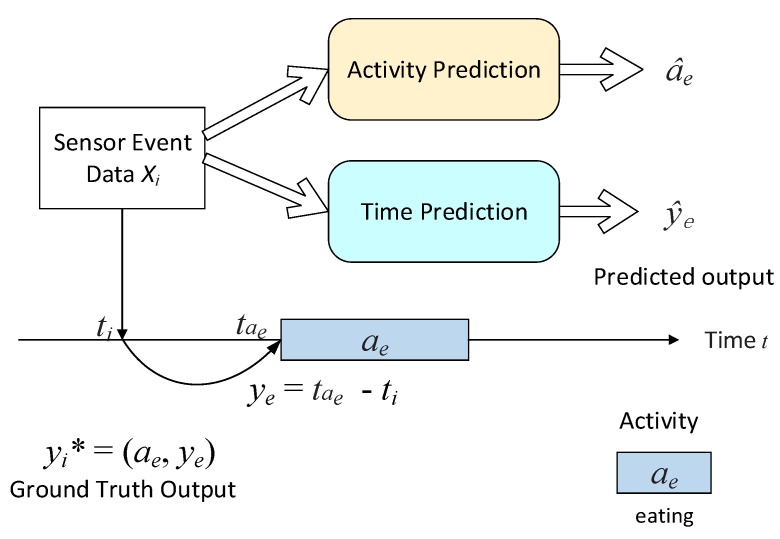
A high-level overview of the multi-task daily activity forecast problem. Given features *X_i_* ∈ *F* extracted from the current sensor event at time *t_i_* as input, the model forecaster needs to forecast daily activity category and the relative occurrence time. In this example, we have the next daily activity category *a_e_* (eating) of the current sensor event and the time *ta_e_* of the event marking the start of daily activity *a_e_*. Therefore, the ground-truth output is y_i_* = (*a_e_*, *y_e_*), where *y_e_* = *ta_e_* - *t_i_* stands for the correct relative occurrence time (minutes) of next daily activity *a_e_*.

Formally, let S = {*I*_1_, *I*_2_, ..., *I_S_*} be a set of sensors installed in the smart home. *A* = {*a*^1^, *a*^2^,..., *a^K^*} is a set of *K* kinds of activities in the dataset, where *a^k^* corresponds to the *k-*th daily activity category. Given a series of samples *F* = {*X*_1_, *X*_2_, ...} extracted from the sensor data as input, the forecast model generates ŷ* = {(*â*_1_, *ŷ*_1_), (*â*_2_, *ŷ*_2_), ...} as output. (*â_i_*, *ŷ_i_*) corresponds to the daily activity *â_i_* ∈ *A* and the relative occurrence time *ŷ_i_* (minutes) of the *i-*th instance. Figure 1 provides an illustration of the multi-task forecast problem. Note that both input features and output predictions correspond to a single sensor event that occurred at specific points in time. 

## 4. Dataset Description

Five publicly available datasets: “MavLab”, “Adlnormal”, “Cairo”, “Tulum2009” and “Aruba” were used to evaluate the proposed approach [52,53]. “MavLab” was published by University of Texas. “Adlnormal”, “Cairo”, “Tulum2009” and “Aruba” were published by the Center for Advanced Studies in Adaptive Systems (CASAS). The kinds of sensors, locations of sensors, categories of involved daily activities are displayed in Table 1. 

**Figure 2:**
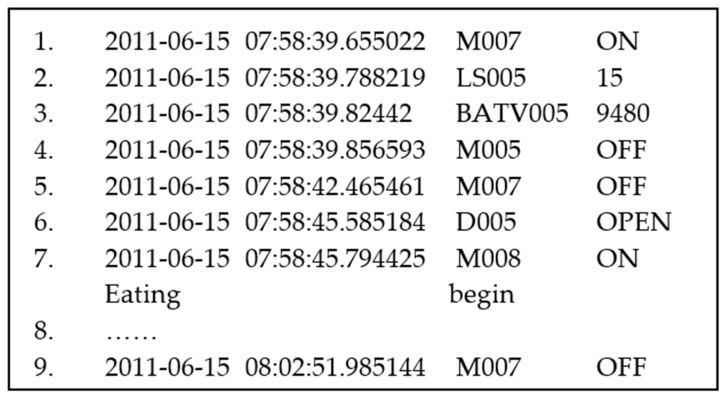
Sequence of sensors activated in chronological order. The end of the seventh sensor event marks the starting of eating activity.

The training data in this paper includes a series of raw sensor events *E* = {*e*_1_, *e*_2_,..., *e_n_*}. As shown in Figure 2, one sensor event *e* is recorded per line, which is expressed as four tuples: *e* = (*D*, *T*, *I*, *R*). *D* and *T* are the date and time when *e* was generated; *I* is the identification of the active sensor, and R is the sensor reading. For example, the sensor event shown in line 7 was generated at 07: 58: 45.794425 on 2011-06-15. The activated sensor is M008 and the reading is ON, and the sensor event labeled the beginning of eating activity.

We further assume the context of the sensor event to calculate the feature vector *X* ∈ *F* for the most recent sensor event *e*. We also establish a multi-task learning forecast model to make multiple forecast outputs have higher test results (such as *F-score* and *NRMSE*).

## 5. Method 

Here, the details of the proposed method are described. The overall the framework involves three steps: initial features generation, model architecture and training.

### 5.1. Initial Features Generation 

For the sequence of sensor events activated by daily activities, the initial feature value *X* of the most recent sensor event for model training is generated by Algorithm 1. Algorithm 1 is divided into two phases. In the first phase (lines 2–5), the temporal features of the most recent sensor events are extracted. In the second phase (lines 6–15), the recent sensor event space features are solved according to the deployed sensor identifications *S*.
**Algorithm 1.** Generate initial features group**Input:***S*, deployed sensor identifications in smart house *E*, A sequence of sensor events activated in the window **Output**: XX←∅;*Te_f_* ←*getFirstSensorEventTime(E); //*Get time of first sensor event *e_f_* in *E*.*Te_l_* ←*getLastSensorEventTime(E); //*Get time of last sensor event *e_l_* in *E*.Δ*t*←*ComputTimeInterval(Te_f_*, *Te_l_)*; //Comput time interval of *e_f,_* and *e_l_.**X*←*X*∪{*Te_f_*, *Te_l_*, Δ*t* };*IS*←{*I|I* ∈ *E(e)*}; //Extract set of *I* in *E***for each***I***in***S***if***I* ∈ *IS* **then***IN*←*ComputNumberSensor(I, IS)*;//Calculate the frequency of sensor *I* at *IS*.*X*←*X*∪{*IN*};**else***X*←*X*∪{0};**end if****end for****return***X*

### 5.2. Model Architecture 

**Figure 3:**
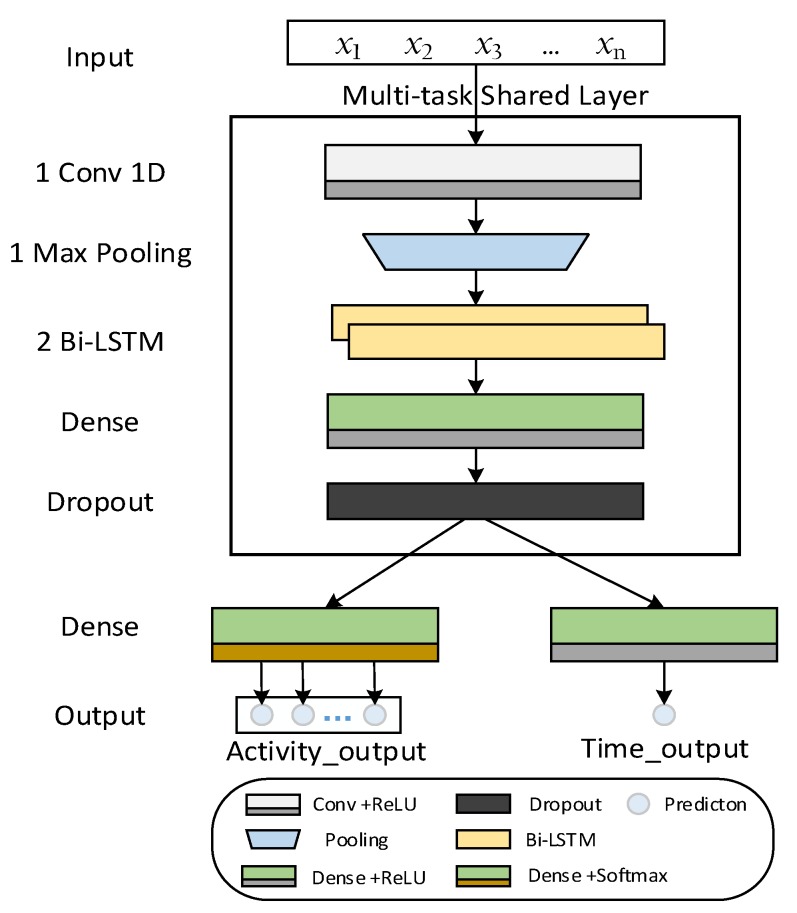
An overview of the multi-task learning architecture in self-boosted forecast model framework.

Multi-task learning based on a neural network is a common method in practical application. Caruana demonstrated early success in this research field [54]. Next, we propose a brief overview of our multi-task architecture. The network architecture mines deeply the input data in both vertical and horizontal direction, which is shown in Figure 3. 

Each task forecasts the next activity information from the most recent sensor event. One is to forecast the next daily activity category of most recent sensor event. The other is to forecast the start time of the daily activity. In the multi-task learning, the two tasks are co-trained to boost the performance of the forecast model.

In particular, the related feature group *X =* {*x*_1_,*x*_2_,...,*x*_n_} is input into the one-dimensional convolutional (Conv1D) layer to extract short-term patterns of the series. The Conv1D layer has 32 one-dimensional filters of size 5. It is followed by the rectified layer unit (ReLU) as a non-linear activation function. A max pooling layer is stacked on top of the convolutional layers. This reduces the latent representation dimension and computation in the network. It is a moving window of size 2, where the maximum value within each window corresponds to the output. The latent-space consists of two shared Bi-LSTM layers of 32 and 16 units. Bi-LSTM helps efficiently discover more high-level features at different time scales, which results in improvement of the forecast performance. The features vector is then passed to shared dense layers, followed by ReLU and dropout (rate 0.2). Finally, the shared features vector is passed to two independent dense layers. One dense layer (activation Softmax) makes classification judgment. And the probability values of category of the next daily activity are output. The other (activation ReLU) makes a regression judgment and outputs the time at which the activity occurred. Task-specific loss functions are then used to learn the weights of the network. 

### 5.3. Training

Network training in this paper is a multiple regression and classification problem. Hence, it involves different loss functions for activity detection and time estimation training.

#### 5.3.1. Category Forecast of Daily Activity

The forecast model of daily activity can estimate the next most possible activity class *a^k^* ∈ *A* for the features in the most recent sensor event, where *A* = {*a^1^*, *a^2^*, ..., *a^K^*}. Therefore, the sparse categorical cross entropy loss function given in (1) is used to train the activity detection task:

(1) $${\tilde{L}}_{A}=-\sum_{i=1}^{N}\sum_{k=1}^{K}a_{i}^{k}\cdot \log({\hat{a}}_{i}^{k})$$

In Equation (1), *a_i_^k^* is the *i-*th sample ground-truth daily activity category, and *â_i_^k^* is the predicted probability of the target daily activity category. The probability values *â_i_^k^* is obtained from the last fully connected layer for the network model.

#### 5.3.2. Occurrence Time Forecast of Daily Activity

For outliers with large differences in the dataset, we use the Huber loss function to avoid the impact of outliers to a certain extent, making training more robust to outlier. The Huber loss function is defined in Equation (2):

(2) $${\tilde{L}}_{T}=\sum_{i=1}^{N}I_{|y_{i}-{\hat{y}}_{i}|\leq \delta }\cdot {\frac{\left(y_{i}-{\hat{y}}_{i}\right)}{2}}^{2}+I_{|y_{i}-{\hat{y}}_{i}|>\delta }\cdot (\delta \cdot |y_{i}-{\hat{y}}_{i}|-\frac{1}{2}\cdot \delta^{2})$$

where *ŷ_i_* is the estimated occurrence time value of the *i-*th sample, *y_i_* is the real value, and *δ* is a Huber loss hyperparameter. The choice of *δ* determines the behavior of the model in dealing with outliers. 

The objective of this paper is to minimize joint losses for all tasks. In particular, the joint loss function *L_full_* is defined by the average weighted loss of all task-specific losses:

(3) $$L_{full}=\frac{1}{2}\cdot (\lambda_{A}\cdot {\tilde{L}}_{A}+\lambda_{T}\cdot {\tilde{L}}_{T})$$

where the weight parameters *λ_A_*, *λ_T_* are determined by the importance of the task in the overall loss. More penalties are imposed for errors on the primary task. Hence, we set the weight to 10 times that of the second task.

In the forecast model of multi-task learning, as shown in Figure 3, two tasks share features and network structure together during iterative training. They are separated at the last fully connected layer. In each iteration of the iterative model, randomly select one task from *M* tasks and update the model according to the task-specific target. Algorithm 2 is repeatedly executed until the maximum epoch number *T* of training models is reached.
**Algorithm 2.** Training algorithm for multi-task forecast model**Input:***F*, Sequence of training datasets for two forecast tasks. y*, the ground-truth output values **Output:**ŷ*, predicted values*P*←0; //initializes model parameters**while***t* ≤ *T* **do****for each** subtask *m* **in** *M**L_m_*←*lossfunction_m_*(*F*); //loss for the *mth* taskΔ*p_m_*←calculatesgradientdescent(*L_m_*);//Adam algorithm is used to calculate Δ*p_m_* gradient descent.**end for**$\Delta p\leftarrow \frac{1}{M}\sum_{i=1}^{M}\Delta p_{i}$;P←getnewparameters(Δp);//update the model parameter P**end while****return**ŷ*

## 6. Evaluation

In this section, several evaluation methods are introduced to evaluate the proposed model. The quality and usefulness of a particular metric will vary with the given problem and the specific evaluation criteria. Therefore, it is necessary to select multiple metrics to verify the effectiveness of different methods.

### 6.1. Classification Evaluation Metrics

Category forecasts of daily activity can be viewed as a type of classification task. If the prediction probability that a sample belongs to the *k-*th class is less than the threshold, the sample is treated as a mislabeled data point. In this case, daily activity forecast model can be evaluated in a variety of ways regarding the type of performance required. The performance indicators based on the classifier were the *Accuracy*, *Recall*, *Precision*, and *F-score*, which are defined in Equations (4), (5), (6) and (7), respectively. *K* is the number of activity labels. *TP_i_* is the number of true positives. *FP_i_* is the number of false positives. *FN_i_* is the number of false negatives. *TN_i_* is the number of true negatives.

(4) $$Accuracy=\frac{\sum_{i=1}^{K}TP_{i}}{\sum_{i=1}^{K}TP_{i}+FP_{i}}$$

(5) $$Recall=\frac{\sum_{i=1}^{K}\frac{TP_{i}}{TP_{i}+FN_{i}}}{K}$$

(6) $$Precision=\frac{\sum_{i=1}^{K}\frac{TP_{i}}{TP_{i}+FP_{i}}}{K}$$

(7) $$F-score=\frac{2\cdot Precision\cdot Recall}{Precision+Recall}$$

### 6.2. Regression Evaluation Metrics

Occurrence time forecast of daily activity can be viewed as a type of regression forecast task. We evaluate the error between the predicted value and the actual value of the task based on the evaluation index of the regression model. The *Mean Absolute Error* (*MAE*), which is defined in Equation (8), provides a measure of the proximity between the predicted output and the real output. Another well-known metric, *Root Mean Squared Error* (*RMSE*), is defined in Equation (9). In Equation (10), the *R-squared* (*R^2^*) provides the variance change of the independent variable to explain the dependent variable.

(8) $$MAE=\frac{1}{N}\cdot \sum_{i=1}^{N}\left|y_{i}-{\hat{y}}_{i}\right|$$

(9) $$RMSE=\sqrt{\frac{1}{N}\cdot \sum_{i=1}^{N}{\left(y_{i}-{\hat{y}}_{i}\right)}^{2}}$$

(10) $$R^{2}=1-\frac{\sum_{i=1}^{N}{\left(y_{i}-{\hat{y}}_{i}\right)}^{2}}{\sum_{i=1}^{N}{\left(y_{i}-\bar{y}\right)}^{2}}$$

where *ӯ* is the mean of the actual values of all the estimated data. 

*RMSE* and *MAE* measures provide the average error in real units (minutes) at different angles. They also quantify the total error rate. Because the variability between activities cannot be used effectively to compare the forecast errors of different activities the evaluation score will be distorted due to the existence of outliers. In these cases, metrics such as *MAE* and *RMSE* do not give an indicative relative error. Therefore, normalized errors *Normalized MAE* (*NMAE*) and *Normalized RMSE* (*NRMSE*) are used. *NMAE* and *NRMSE* are defined in Equations (11) and (12). *y_ma_*_x_ and *y_min_* are the actual maximum and minimum values for all test instances. This metric is usually applied to different datasets. Normalization indexes can easily compare the results of different sets. There is no clear unified standard for normalization factors, so they cannot be used to evaluate the actual magnitude of the error:

(11) $$NMAE=\frac{\frac{1}{N}\cdot \sum_{i=1}^{N}\left|y_{i}-{\hat{y}}_{i}\right|}{\bar{y}}$$

(12) $$NRMSE=\frac{\sqrt{\frac{1}{N}\cdot \sum_{i=1}^{N}{\left(y_{i}-{\hat{y}}_{i}\right)}^{2}}}{y_{\max}-y_{\min}}$$

## 7. Experiments and Discussion

### 7.1. Experimental Setup

Five public datasets “MavLab”, “Adlnormal”, “Cairo”, “Tulum2009” and “Aruba” are used to evaluate the proposed model. The first dataset is the MavLab dataset collected in the MavHome testbed at the University of Texas (Arlington, TX, USA) [52]. Others are collected from CASAS smart home and provided by the Washington State University [53]. Details of the five datasets are shown in Table 2. 

We use sliding windows to train and test the proposed model. This method uses a fixed-length sliding window and moves it across the datasets to segment time series data. The last event in the window is taken as the most recent sensor event. The initial feature group of the event in the window is extracted using Algorithm 1. Then the window is moved forward the specified step size (number of sensor events), and the process is repeated. Finally, the sample data are randomly divided into training (60%), verification (20%) and testing (20%). Table 3 provides some specific parameter settings during model training.

We evaluate two recurring factors that affect the forecaster performance. The first is the weight setting of the joint loss function of multi-task. It uses the loss-weighted sum method of different tasks. The second is the size of the training window. It takes into account the impact on performance of information about the context of recent sensor events. Compared with the best single-task learning models, the proposed model achieves better performance.

### 7.2. Comparison of Different Loss Weight

Table 4, Table 5 and Table 6 show the forecast performance of the multi-task forecast method proposed in this paper. We compare the evaluation metrics of the classification task and regression task of three groups of loss weights on three datasets (Cairo, Tulum2009 and Aruba). 1000, 2000, 3000, 4000 and 5000 are assigned to the training window size to evaluate the learning capabilities of the models. The best results are highlighted in bold underline.

The deep learning forecast model under the third set of loss weight values (*λ_A_* = 1, *λ_T_* = 0.1) is generally better than the two groups. This explains that under the optimal weight, multi-task can better coordinate training and promote each other to improve generalization skills. For the Cairo dataset, performance of the third group is significantly higher than the other two groups in terms of *F-score* and *NPMSE* when 1000, 3000, 4000, 5000 are assigned to window size. The *F-score* of the category forecast of daily activity are 0.9459, 0.9451, 0.9255 and 0.9405, respectively. There are 3.11%, 4.5%, 1.46%, and 2% improvements over the better outcomes of the first two groups. For occurrence time forecasts of daily activity, the *NRMSE* values increase by at least 6.57% compared with the results in the first two groups. For the Tulum2009 dataset, the third set beat the other groups when 1000, 4000, 5000 are assigned to window size. *F-score* can increase by 8.52%, 5.86%, and 1.5%. *NPMSE* values increase by 3.79%, 1.8%, and 7.15%. The Aruba dataset also gets the same result pattern, which outperform the other groups when 2000, 4000 are assigned to window size. Although it lags behind other groups in the rest of the window, other metrics of the model still perform well. Therefore, seeking better weight settings plays an important role in improving the performance of the model.

**Figure 4:**
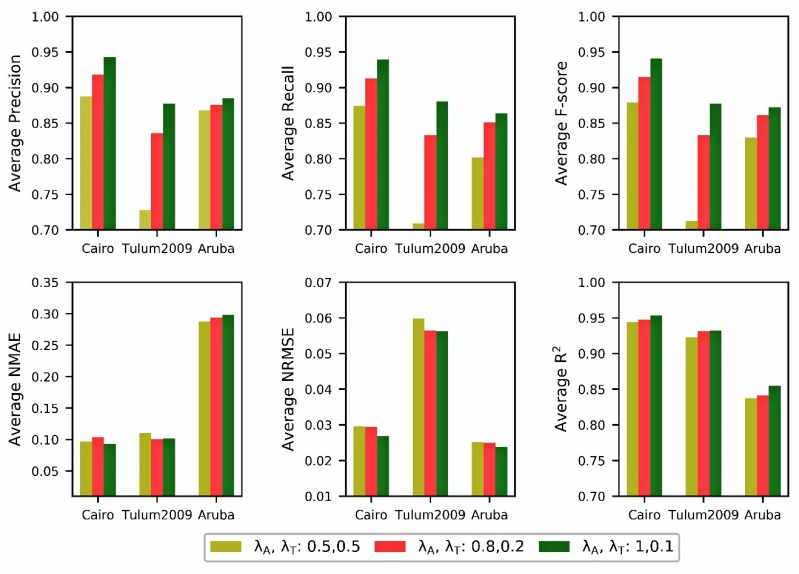
Performance evaluation of the multi-task forecast model with different loss weights on three datasets. The first row represents the average measures of category forecast of daily activity (Average *Precision*, Average *Recall,* Average *F-score*). The other row is the average measures of occurrence time forecast of daily activity (Average *NMAE*, Average *NRMSE*, Average *R^2^*).

Furthermore, to promote the performance comparison of the model in the window size, the six evaluation metrics of all weight settings are averaged in this paper. Figure 4 shows that the forecast model performs better under the third set of weights than the other two sets. In particular, category forecast of daily activity achieves significant improvements in the classification evaluation metrics.

### 7.3. Training Window Size

Based on the results in Table 4, Table 5 and Table 6, the performance of a multi-task forecast model is partially dependent on the size of the used training window. Therefore, we perform relevant verification in Table 7, Table 8 and Table 9. In the tests, the relatively small training window can obtain a sufficient number of test points to calculate evaluation indexes. Although overfitting of the model is prevented, this results in the lack of information about daily activity. Oversize the training window can result in performance degradation. Therefore, sliding windows of 1000, 2000, 3000, 4000 and 5000 events are used to determine the effect of training window size on performance. For all tests, each iteration moves the window forward 20 events.

Table 7, Table 8 and Table 9 show the test results of six average metrics of the three weight values. These tables indicate that the optimal training window size may vary between datasets and activities. For the Cairo dataset, the overall evaluation value of the forecast model is not stand out in each window size. Therefore, model performance is not sensitive to window size. For the Tulum2009 dataset, the model with the highest performance is the model with a sliding window size of 3000. 

For example, the average *F-score* in this test has the best effect of 0.8409. The average *NRMSE* value also improves by at least 1.8% compared with the performance of other tests. The Aruba dataset also beat the other tests in 2000. The average *Recall* is 0.8477, which is slightly behind the results of the 4000 window size model. But the model still performs best in other average evaluation indicators in 2000. The optimal window size varies in different datasets.

### 7.4. Daily Activity Forecast for Multi-Task and Single-Task

To check the effectiveness of the multi-task learning model for each task in daily activity forecast, we select the benchmark method to compare the performance of each forecast task. In addition, based on the test results of the above two parts, the loss weights for multi-task learning are all set as *λ_A_* = 1, *λ_T_* = 0.1.

Firstly, for category forecasts of daily activity, we compare the proposed model (multi-task CNN+Bi-LSTM) with SPADE [14], LSTM [22] and CNN+Bi-LSTM models in two datasets (Adlnormal, MavLab). The experimental results are shown in Table 10. Compared with the benchmark method, the forecast performance of the proposed method is significantly improved. At the same time, the *Accuracy* of the two datasets are 0.9323 and 0.8673, respectively. In particular, it achieve at least 2.93%, 2.22% improvements over other benchmark models. This shows that the proposed model has good performance in task of category forecast of daily activity.

**Figure 5:**
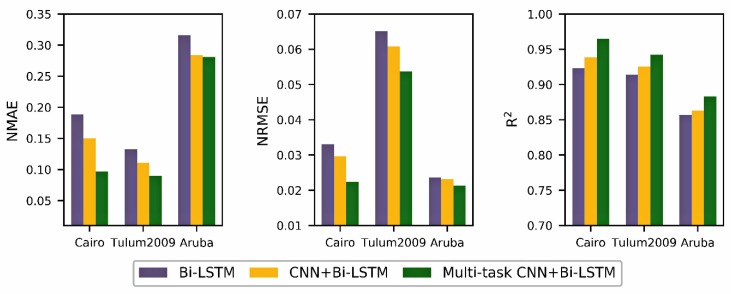
Performance comparison of the different daily activity occurrence time forecast model on three datasets.

Secondly, for occurrence time forecast of daily activity, the proposed model is compared with other single-task learning models to check the generalization ability of models. We use three datasets (Cairo, Tulum2009 and Aruba) in the three evaluation metrics (*NMAE*, *NRMSE* and *R^2^*) mentioned above. The baseline methods include Bi-LSTM and CNN+Bi-LSTM. The specific results are shown in Table 11 and Figure 5. 

For the Cairo dataset, the proposed model achieves 0.0971, 0.0224, and 0.965 at *NMAE*, *NRMSE* and *R^2^*. It has improvements of 35.44%, 24,49%, and 2.71% over the best benchmark. For the Tulum2009 dataset, this proposed model is also significantly higher than other benchmark methods. There are at least 18.76%, 11.68%, and 1.69% improvements in the three metric settings. The Aruba dataset also has the same result pattern, which is better than the previous two single-task learning methods. It demonstrates that the proposed model can effectively forecast occurrence time of a given daily activity.

### 7.5. Discussion

We discuss in this section a few crucial observations from our experiments. As shown in Figure 4, all tests benefit from setting appropriate weights for different loss functions. This gain may be mainly due to the fact that the loss of the classification task and regression task is not a magnitude. The rate of gradient descent is not consistent. Thus, setting different loss weights can balance them to some extent.

We also analyze the effect of the size of the sliding window on the model. The optimal window size is different for different datasets. We believe that these differences may be caused by factors such as the type of sensor used in the dataset and the relationship between activities and sensor events. Furthermore, the selection of window size must balance the need for a sufficient number of events in the training window, and the need for the number of samples for model training and performance analysis. 

The performance of our multi-task learning model is better than that of the single-task learning model on multiple datasets. This may be due to the fact that residents perform certain daily activities at fixed times. For example, the activity “work” might start at a set time. Or this is a habit of residents who start performing activities such as “sleep” and “cook” at a particular time. Therefore, there is a special correlation between the daily activities and their occurrence time. Then, multi-task learning technique may use this potential information to improve the results of these two forecast tasks. Moreover, variations between datasets also do not impact the predictions very much, which allows daily activity forecast model for multi-task learning to be applied in a variety of situations.

We note that the weight values of the loss function are manually adjusted in this paper, but the selected values are not necessarily the most appropriate. Consequentially, it may be necessary to perform further studies to automatically select the more appropriate loss weights. Besides, the forecast model needs to be further improved to dig deeper correlation information between daily activity forecast tasks.

## 8. Conclusions

We have conducted a comprehensive study on forecasting of daily activities in a smart home. To address the problem of the traditional methods that tend to lose potential information between forecast tasks, we proposed a forecast model based on multi-task learning. The results showed that the performance was highly dependent on the choice of appropriate loss weights and the optimal window size required for forecast model was determined by the characteristics of datasets. Five distinct datasets were used to evaluate the proposed model. The experimental results showed that compared with the state-of-the-art single-task learning models, the proposed model achieved the best performance.

## Figures and Tables

**Table 1:** Datasets description.

Locations of Sensors	Kinds of Sensors	Daily Activity Categories
“Bedroom”	“Motion sensors”	“Sleep”
		“Breakfast”
“Office”		“Leave_home”
	“Temperature sensors”	“Work_in_office”
“Kitchen”		“Lunch”
		“Dinner”
“Dining room”	“Door sensors”	“Wash_Dishes”
		“Bed_to_toilet”
“Bathroom”	“Light sensors”	“Enter_Home”
		“Watch_TV”
_	_	_

**Table 2:** CASAS smart home datasets involve sensors, sensor events and daily activities.

Dataset	Residents and Pets/Participants	Number of Sensors	Daily Activity Categories	Measurement Time	Sensor Events
MavLab	6 participants	51	10	19 days	3015
Adlnormal	20 participants	25	5	13 days	6425
Cairo	2 residents and 1 pet	32	13	57 days	726534
Tulum2009	2 residents	20	10	84 days	486912
Aruba	1 resident	39	11	90 days	725530

**Table 3:** Parameter Interpretation of Model Training.

Configuration Name	Parameter Interpretation
Hyperparameter of Huber loss	*δ←*1
Gradient descent algorithm	AdamOptimizar
Learning rate	1e-3
Batch size	200
Epoch number	*T←*500

**Table 4:** The Cairo dataset uses six evaluation metrics (*Precision*, *Recall*, *F-score*, *NMAE*, *NRMSE* and *R-squared* (*R^2^*)) for comparison under different loss weights. The best performing tests in each metric are shown in underline.

Loss Weight *λ_A_*, *λ_T_*	Training Window Size	Metrics					
		*Precision*	*Recall*	*F-score*	*NMAE*	*NRMSE*	*R2*
0.5, 0.5	1000	0.8666	0.8565	0.8598	0.1059	0.0292	0.9406
	2000	0.8941	0.8834	0.8873	0.0956	0.0279	0.9457
	3000	0.9046	0.8776	0.8862	0.0837	0.0303	0.9436
	4000	0.8897	0.8798	0.8832	0.0969	0.0299	0.9517
	5000	0.8823	0.8749	0.8768	0.1012	0.0306	0.9377
0.8, 0.2	1000	0.9266	0.9097	0.9174	0.1049	0.0279	0.9599
	2000	0.9177	0.9186	0.9177	0.1079	0.0292	0.9406
	3000	0.9116	0.899	0.9044	0.1013	0.031	0.9408
	4000	0.9171	0.9086	0.9122	0.1034	0.0289	0.9547
	5000	0.9181	0.9275	0.9222	0.1014	0.0301	0.9399
1, 0.1	1000	0.9478	0.9441	0.9459	0.0971	0.0224	0.965
	2000	0.9469	0.9453	0.9458	0.0974	0.0291	0.941
	3000	0.9448	0.946	0.9451	0.0849	0.0288	0.9494
	4000	0.9312	0.9223	0.9255	0.0999	0.027	0.9606
	5000	0.9414	0.9405	0.9405	0.0845	0.0271	0.9514

**Table 5:** The Tulum2009 dataset uses six evaluation metrics (*Precision*, *Recall*, *F-score*, *NMAE*, *NRMSE* and *R-squared* (*R^2^*)) for comparison under different loss weights. The best performing tests in each metric are shown in underline.

Loss Weight *λ_A_*, *λ_T_*	Training Window Size	Metrics					
		*Precision*	*Recall*	*F-Score*	*NMAE*	*NRMSE*	*R2*
0.5, 0.5	1000	0.6639	0.6782	0.6627	0.1286	0.0625	0.9097
	2000	0.7005	0.6772	0.6807	0.1126	0.0648	0.9146
	3000	0.7618	0.7518	0.7543	0.1063	0.0536	0.942
	4000	0.7789	0.7316	0.7495	0.1047	0.0571	0.9314
	5000	0.7324	0.7071	0.7138	0.099	0.0611	0.9169
0.8, 0.2	1000	0.79	0.7839	0.7837	0.101	0.0554	0.9292
	2000	0.8359	0.8453	0.8402	0.0963	0.0517	0.9457
	3000	0.8598	0.8619	0.8603	0.0934	0.0565	0.9358
	4000	0.8517	0.8453	0.8448	0.1056	0.0553	0.9357
	5000	0.8426	0.8305	0.8353	0.1054	0.0629	0.9116
1, 0.1	1000	0.8499	0.8529	0.8505	0.11	0.0533	0.9344
	2000	0.8994	0.878	0.8868	0.1126	0.0613	0.9236
	3000	0.9108	0.9066	0.9081	0.0901	0.0537	0.9421
	4000	0.8877	0.9019	0.8943	0.0912	0.0543	0.9378
	5000	0.838	0.8623	0.8478	0.1028	0.0584	0.924

**Table 6:** The Aruba dataset uses six evaluation metrics (*Precision*, *Recall*, *F-score*, *NMAE*, *NRMSE* and *R-squared* (*R^2^*)) for comparison under different loss weights. The best performing tests in each metric are shown in underline.

Loss Weight *λ_A_*, *λ_T_*	Training Window Size	Metrics					
		*Precision*	*Recall*	*F-score*	*NMAE*	*NRMSE*	*R2*
0.5, 0.5	1000	0.8761	0.8024	0.8345	0.2802	0.024	0.8468
	2000	0.8765	0.7993	0.8321	0.2868	0.023	0.8603
	3000	0.8477	0.8067	0.8226	0.299	0.0318	0.7735
	4000	0.8597	0.8004	0.8253	0.2989	0.0255	0.8357
	5000	0.8793	0.7989	0.8337	0.2741	0.0215	0.8706
0.8, 0.2	1000	0.8815	0.8345	0.8556	0.2939	0.0245	0.841
	2000	0.8956	0.8686	0.8791	0.2743	0.0239	0.8532
	3000	0.8673	0.8672	0.8664	0.2919	0.0258	0.8506
	4000	0.8588	0.8412	0.848	0.3123	0.0243	0.8506
	5000	0.8736	0.8432	0.8566	0.2966	0.026	0.8097
1, 0.1	1000	0.9098	0.8657	0.8838	0.2998	0.0243	0.8425
	2000	0.9045	0.8751	0.8895	0.2808	0.0213	0.8832
	3000	0.8508	0.8745	0.8614	0.3089	0.0274	0.832
	4000	0.8702	0.8697	0.868	0.2949	0.0234	0.8605
	5000	0.8899	0.8335	0.8575	0.3053	0.0227	0.8552

**Table 7:** Comparison test of six the average metrics of the training window size (in number of events) in Cairo dataset. The best performing tests in each metric are shown in underline. All tests move 20 events per iteration.

Training Window Size	Average Metrics					
	*Precision*	*Recall*	*F-score*	*NMAE*	*NRMSE*	*R2*
1000	0.9137	0.9034	0.9077	0.1026	0.0265	0.9552
2000	0.9196	0.9158	0.9169	0.1003	0.0287	0.9424
3000	0.9203	0.9075	0.9119	0.0900	0.03	0.9446
4000	0.9127	0.9036	0.907	0.1001	0.0286	0.9557
5000	0.9139	0.9143	0.9132	0.0957	0.0293	0.943

**Table 8:** Comparison test of six the average metrics of the training window size (in number of events) in Tulum2009 dataset. The best performing tests in each metric are shown in underline. All tests move 20 events per iteration.

Training Window Size	Average Metrics					
	*Precision*	*Recall*	*F-score*	*NMAE*	*NRMSE*	*R2*
1000	0.7679	0.7717	0.7656	0.1132	0.0571	0.9244
2000	0.8119	0.8002	0.8026	0.1072	0.0593	0.9280
3000	0.8441	0.8401	0.8409	0.0966	0.0546	0.94
4000	0.8394	0.8263	0.8295	0.1005	0.0556	0.935
5000	0.8043	0.8	0.7990	0.1024	0.0608	0.9175

**Table 9:** Comparison test of six the average metrics of the training window size (in number of events) in Aruba dataset. The best performing tests in each metric are shown in underline. All tests move 20 events per iteration.

Training Window Size	Average Metrics					
	*Precision*	*Recall*	*F-score*	*NMAE*	*NRMSE*	*R2*
1000	0.8891	0.8342	0.858	0.2913	0.0243	0.8434
2000	0.8922	0.8477	0.8669	0.2806	0.0227	0.8656
3000	0.8553	0.8495	0.8501	0.2999	0.0283	0.8187
4000	0.8629	0.8371	0.8471	0.3020	0.0244	0.8489
5000	0.8809	0.8252	0.8493	0.2920	0.0234	0.8452

**Table 10:** *Accuracy* comparison results for the models of category forecast of daily activity. The best performing tests in each metric are shown in underline. The two dataset tests are listed by specific sliding window sizes. The sliding window sizes are 50 and 20, respectively, while the number of moves per iteration is 5, 1, respectively.

Method	Dataset	
	Adlnormal	MavLab
SPADE	0.8047	0.8411
LSTM	0.9030	0.8451
CNN+Bi-LSTM	0.8964	0.8401
Multi-task CNN+Bi-LSTM	0.9323	0.8673

**Table 11:** *NMAE*, *NRMSE* and *R^2^* comparison results for the models of occurrence time forecast of daily activity. The best performing tests in each metric are shown in underline. The three dataset tests are listed by specific sliding window sizes, where the sliding window sizes are 1000, 3000 and 2000, respectively. All tests move 20 events per iteration.

Method	Metrics	Dataset		
		Cairo	Tulum2009	Aruba
Bi-LSTM	*NMAE*	0.1883	0.1323	0.3158
	*NRMSE*	0.0331	0.0652	0.0236
	*R2*	0.9233	0.9142	0.8569
CNN+Bi-LSTM	*NMAE*	0.1504	0.1109	0.2852
	*NRMSE*	0.0296	0.0608	0.0231
	*R2*	0.9379	0.9252	0.8632
Multi-task CNN+Bi-LSTM	*NMAE*	0.0971	0.0901	0.2808
	*NRMSE*	0.0224	0.0537	0.0213
	*R2*	0.9650	0.9421	0.8832
